# Supplementary material for: Nesting of multiple polyhedral plasmonic nanoframes into a single entity
Source: Nat Commun. 2022 Aug 4;13:4544. doi: 10.1038/s41467-022-32261-9 (PMC9352762; doi:10.1038/s41467-022-32261-9)
Supplement: Supplementary file 1 — Supplementary Information [file 41467_2022_32261_MOESM1_ESM.pdf]

Supplementary Information

Nesting of multiple polyhedral plasmonic nanoframes into a single entity

Sungjae Yoo[1,2]†, Jaewon Lee[2]†, Hajir Hilal[2], Insub Jung[2,3], Woongkyu Park[4], Joong Wook Lee[5], Soobong Choi[6], and Sungho Park*[2]

[1]Research Institute for Nano Bio Convergence, Sungkyunkwan University; Suwon, 16419, Republic of Korea

[2]Department of Chemistry, Sungkyunkwan University; Suwon, 16419, Republic of Korea

[3]Institute of Basic Science, Sungkyunkwan University; Suwon, 16419, Republic of Korea

[4]Medical & Bio Photonics Research Center, Korea Photonics Technology Institute (KOPTI); Gwangju, 61007, Republic of Korea

[5]Department of Physics and Optoelectronics Convergence Research Center, Chonnam National University; Gwangju, 61186, Republic of Korea

[6]Department of Physics, Incheon National University; Incheon, 22012, Republic of Korea.

*Corresponding author. Email: spark72@skku.edu

†These authors contributed equally: Sungjae Yoo, Jaewon Lee

This pdf file includes :

Materials and instruments
Supplementary Fig. 1 to 22
Supplementary Table 1 to 6
Supplementary References 1 to 13

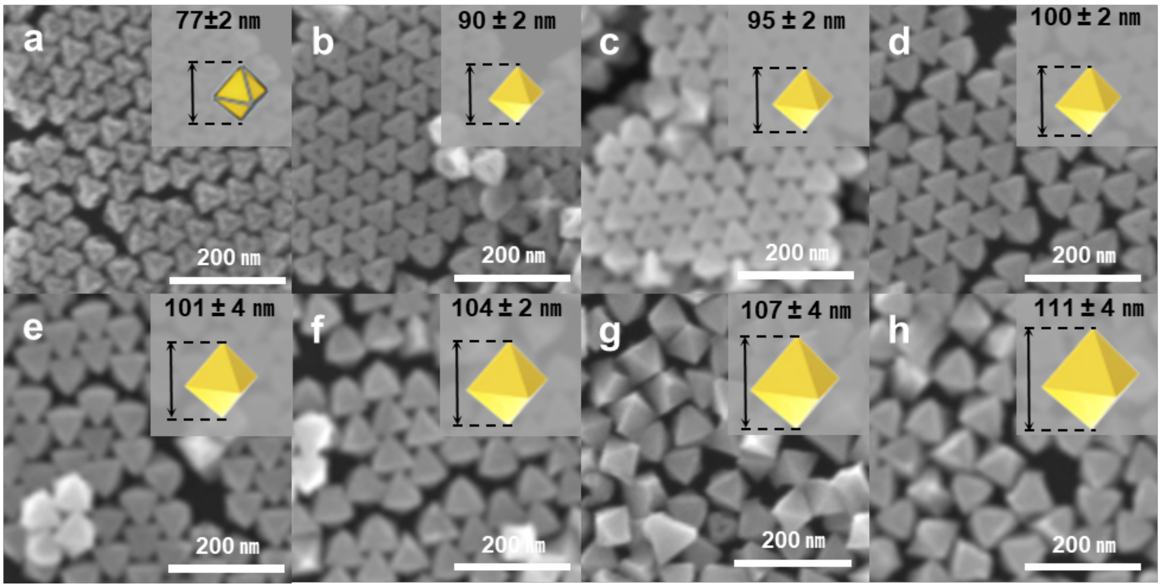

Supplementary Fig. 1 | Shape evolution of Au nanoparticles during well-faceted overgrowth of Au on 1st-Au-O-NPs. (a to h) Monitoring the shape evolution after well-faceted overgrowth of Au on the 1st-Au@Pt-O-NPs through SEM images.

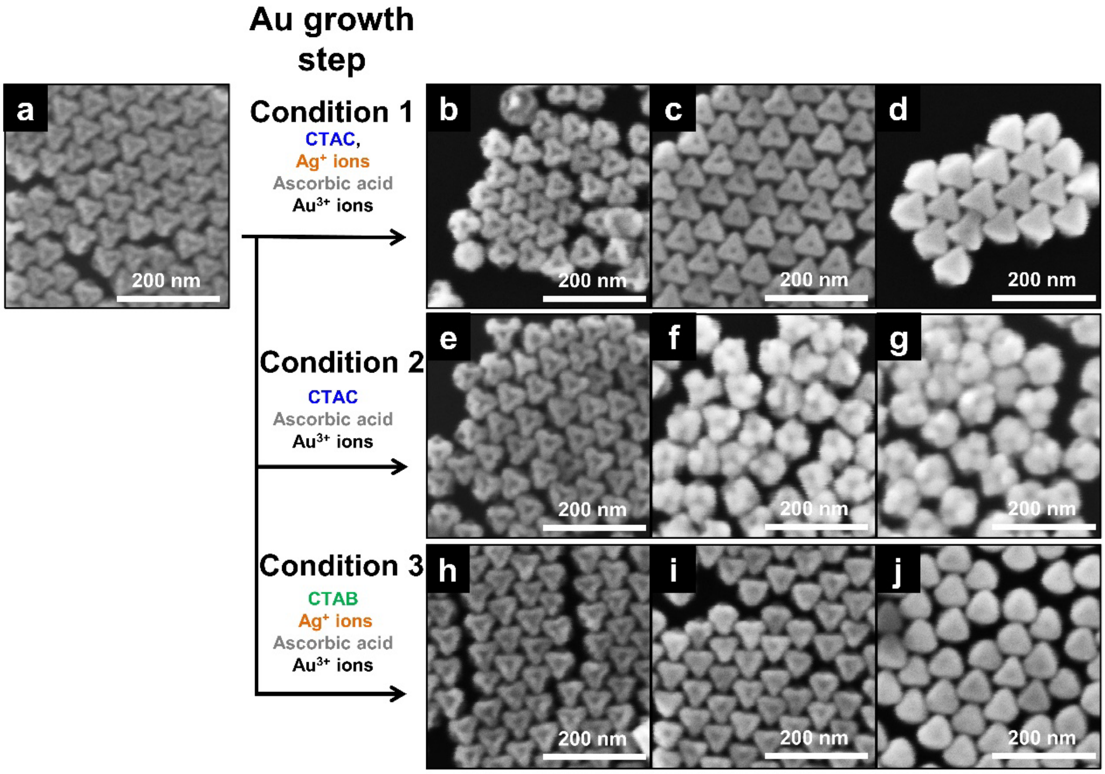

Supplementary Fig. 2 | Investigation of mechanism of the well-faceted overgrowth of Au. (a) SEM image of 1st-Au@Pt-O-NPs. SEM images of the resulting Au nanostructure after well-faceted overgrowth of Au with increasing amounts of Au^{3+} ions (b to d) in CTAC environment with Ag^{+} ions, (e to g) in CTAC environment without Ag^{+} ions, and (h to j) in CTAB environment with Ag^{+} ions.

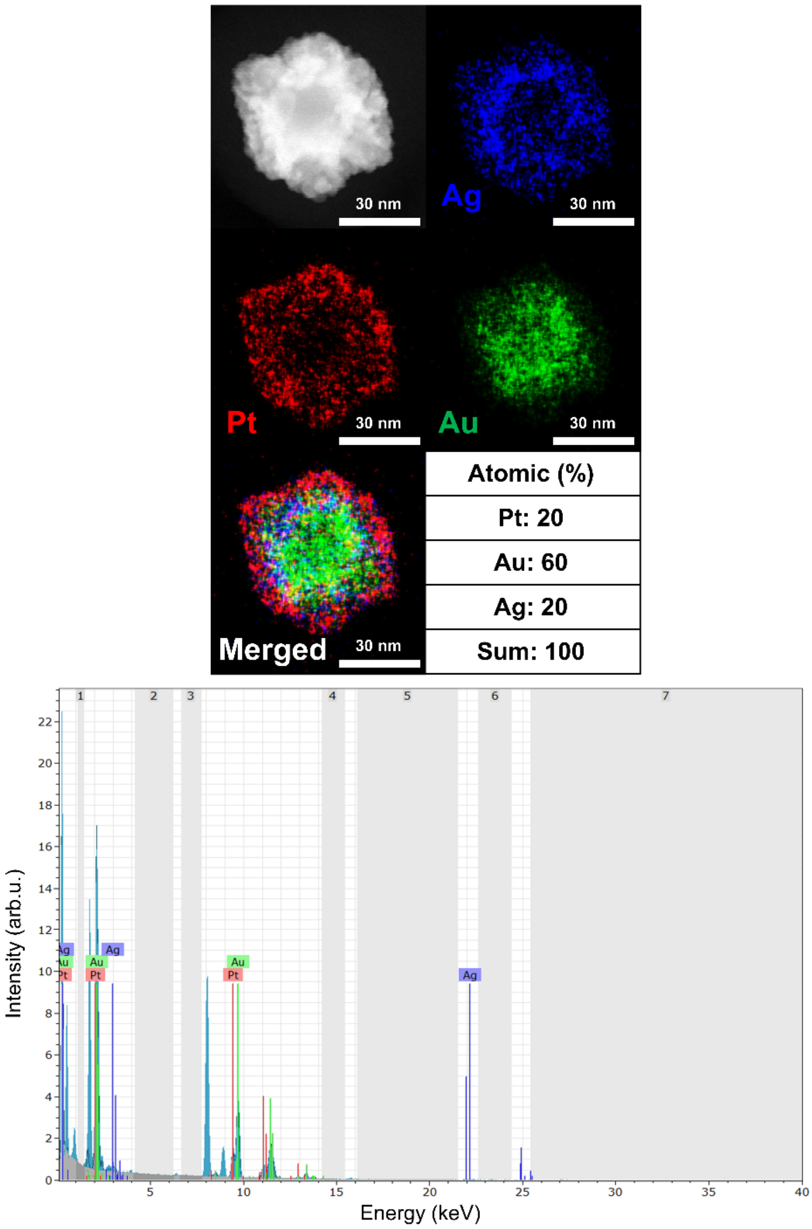

Supplementary Fig. 3 | Analysis of the elemental distribution of Ag-deposited 1st-Au@Pt-O-NP. Distribution of metal elements of Ag-deposited 1st-Au@Pt-O-NP were investigated using energy dispersive X-ray spectroscopy (EDS) in scanning transmission electron microscopy (STEM). Each color used in EDS mapping images (blue for Ag, red for Pt, and green for Au) correspond to the EDS spectra of each element.

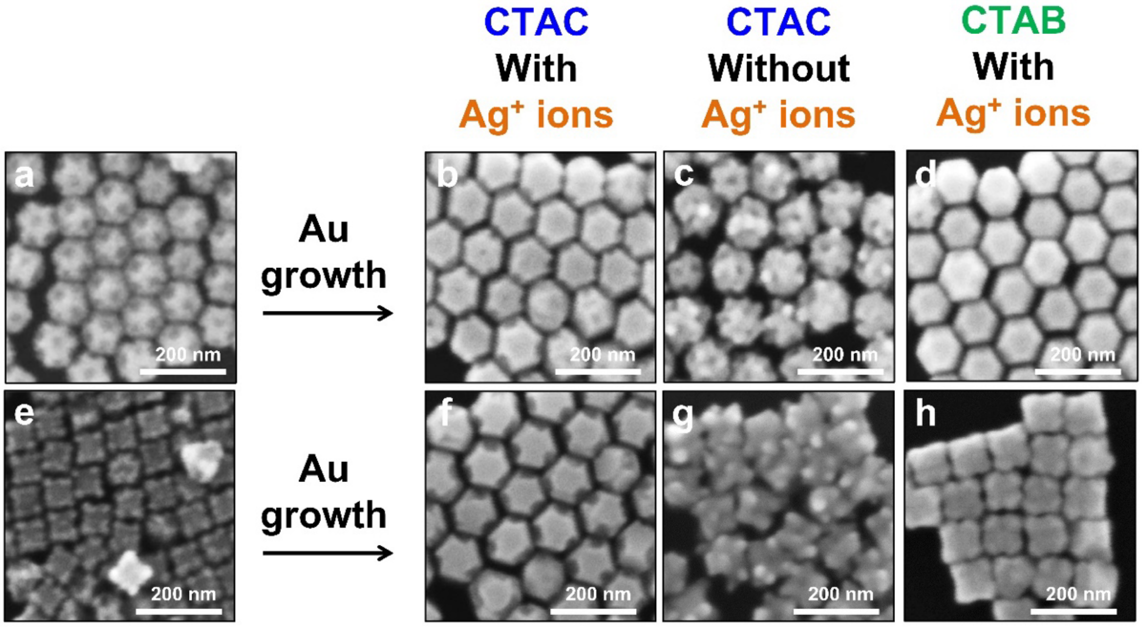

Supplementary Fig. 4 | Applicability of well-faceted overgrowth of Au for differently shaped Au@Pt NPs. (a and e) SEM images of 1st-Au@Pt-TO-NPs and 1st-Au@Pt-C-NPs. (b to h) SEM images of the resulting Au nanostructures after well-faceted overgrowth of Au on Au@Pt nanoparticles (b and f) in CTAC environment with Ag^+ ions, (c and g) in CTAC environment without Ag^+ ions, and (d and h) in CTAB environment with Ag^+ ions.

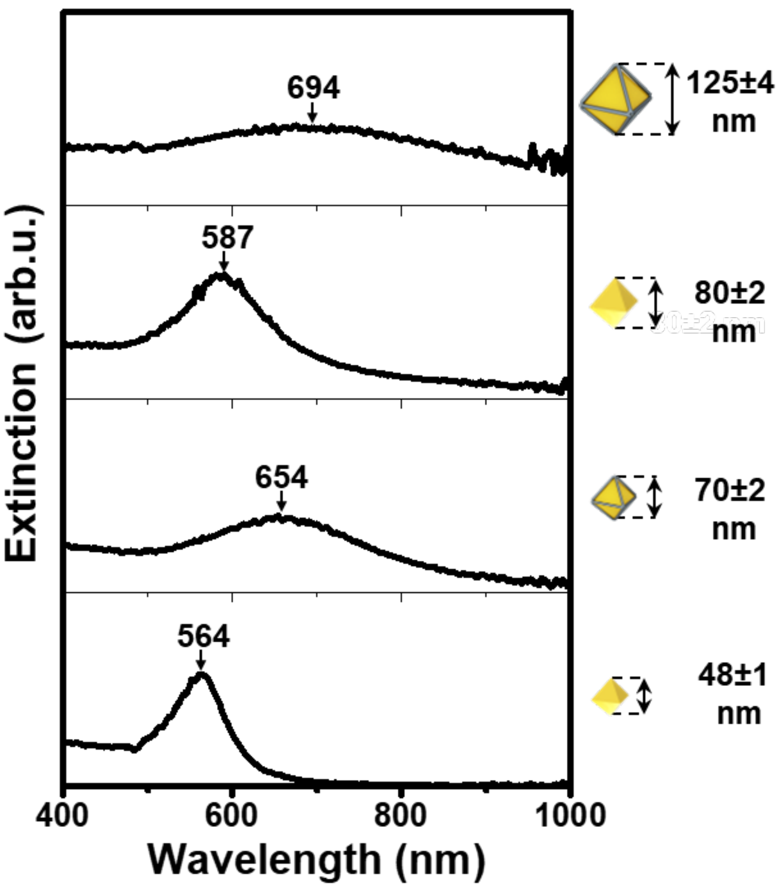

Supplementary Fig. 5 | Monitoring the shape evolution from 1st-Au-O-NPs to 2nd-Au@Pt -O-Nps through UV-vis spectroscopy. Corresponding UV-vis spectrum to each synthetic steps for 2nd-Au@Pt-O-NPs were shown in this panel.

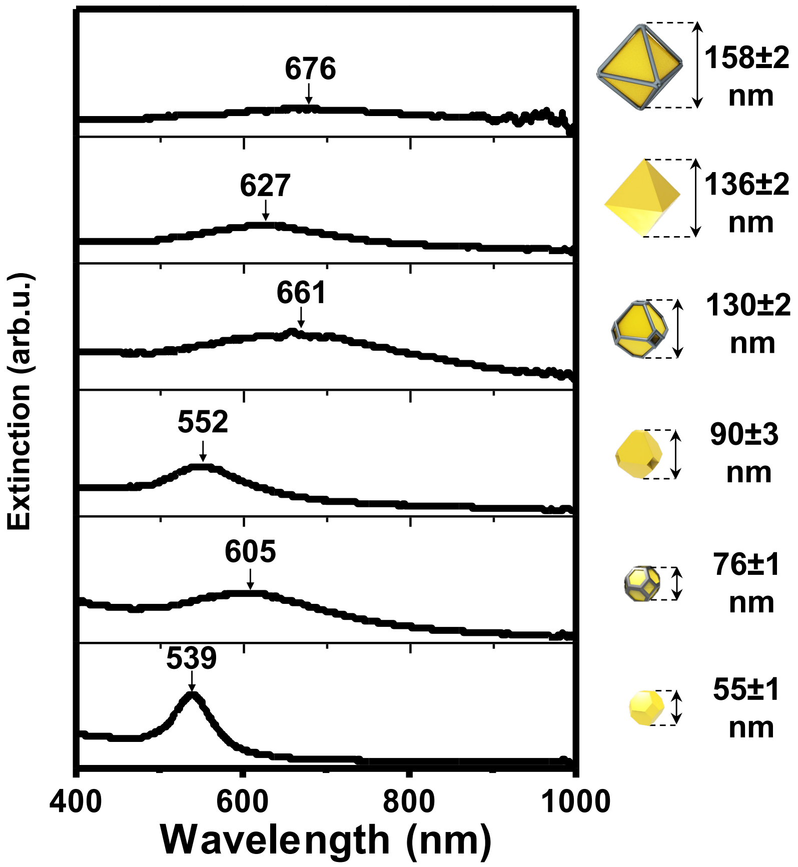

Supplementary Fig. 6 | Monitoring the shape evolution from 1st-Au-TO-NPs to 3rd-Au@Pt-O-NPs through UV-vis spectroscopy. Corresponding UV-vis spectrum to each synthetic steps for 3rd-Au@Pt-O-NPs were shown in this panel.

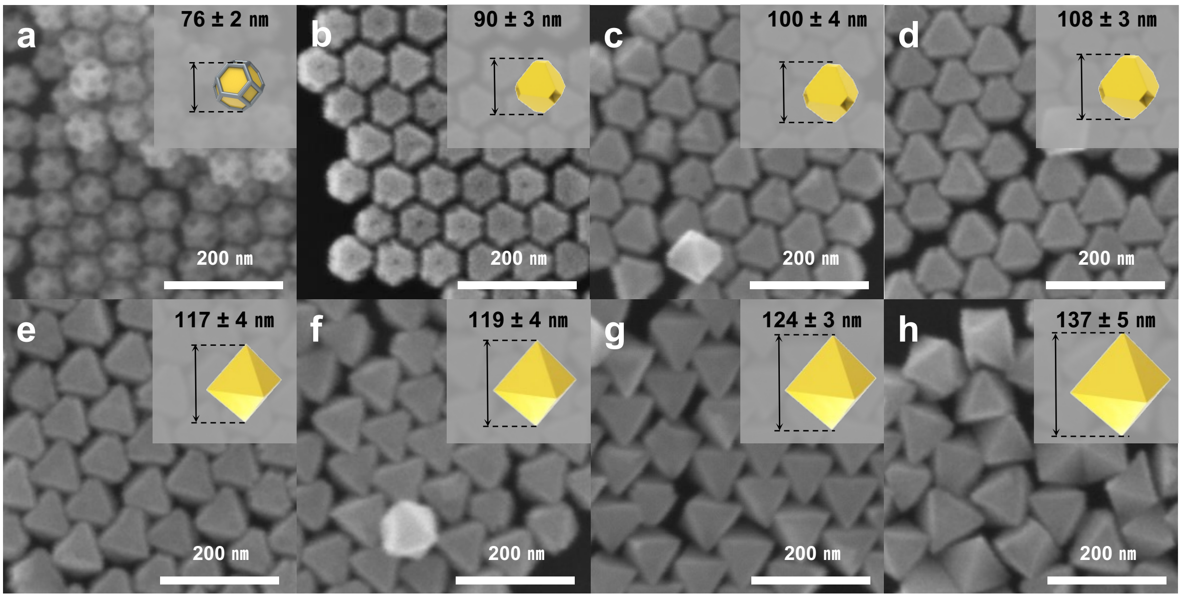

Supplementary Fig. 7 | Shape evolution of Au nanoparticles during well-faceted overgrowth of Au on 1st-Au-TO-NPs. (a to h) Monitoring the shape evolution after well-faceted overgrowth of Au on 1st-Au@Pt-TO-NPs through SEM images.

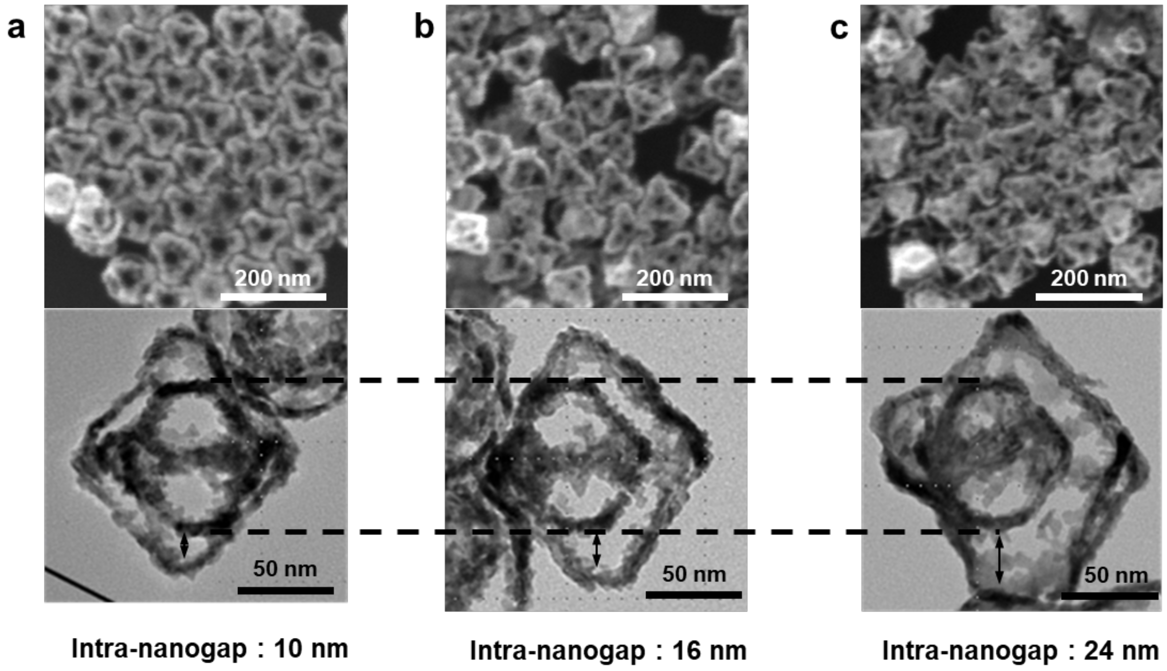

Supplementary Fig. 8 | Controlling the intra-nanogap distance of 2nd-Pt-TO:O-NFs by tailoring the degree of well-faceted overgrowth of Au. (a to c) Low-magnification SEM and zoomed-in TEM images of 2nd-Pt-TO:O-NFs with intra-nanogap distances of (a) 10 nm, (b) 16 nm, and (c) 24 nm.

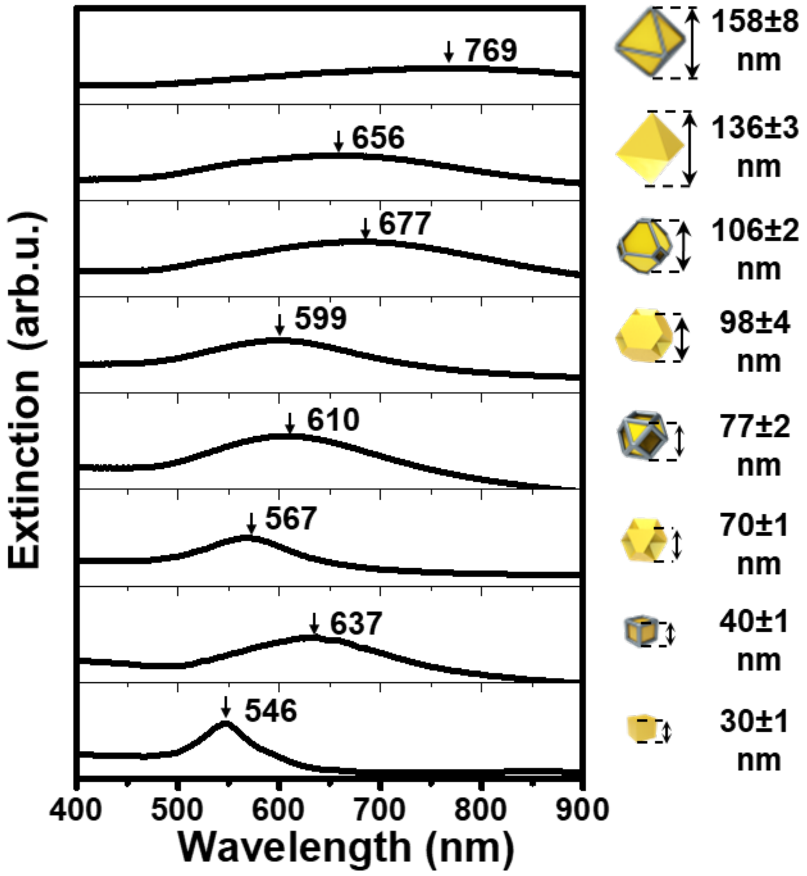

Supplementary Fig. 9 | Monitoring the shape evolution from 1st-Au-C-NPs to 4th-Au@Pt-O-NPs through UV-vis spectroscopy. Corresponding UV-vis spectrum to each synthetic steps for 4th-Au@Pt-O-NPs were shown in this panel.

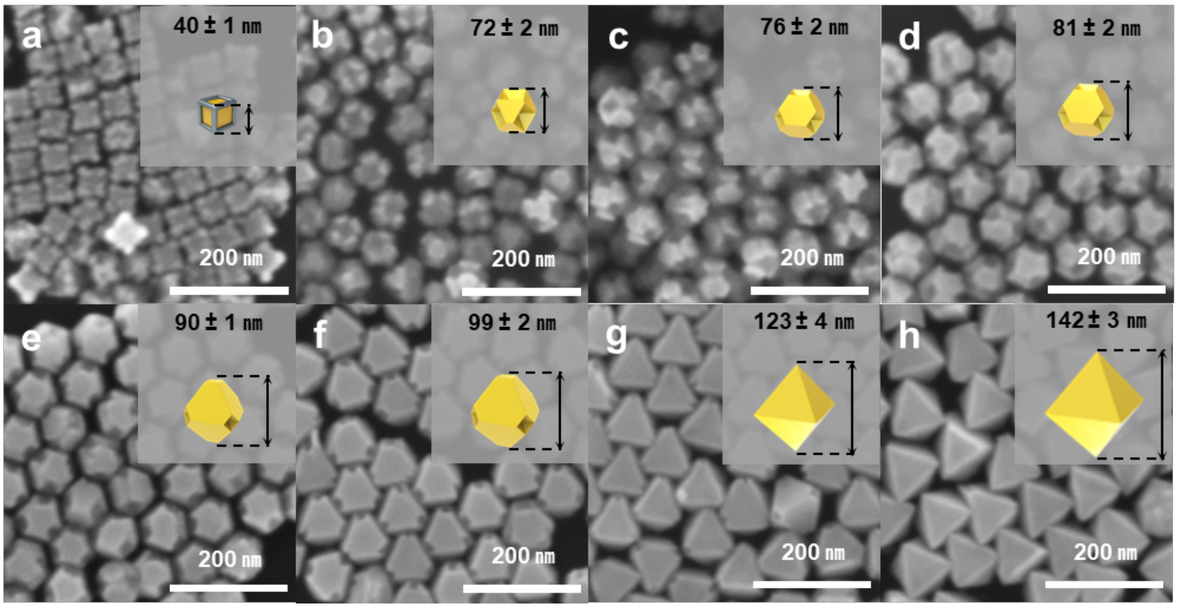

Supplementary Fig. 10 | Shape evolution of Au nanoparticles during well-faceted overgrowth of Au on 1st-Au-C-NPs. (a to h) Monitoring the shape evolution after well-faceted overgrowth of Au on the 1st-Au@Pt-C-NPs as illustrated by SEM images.

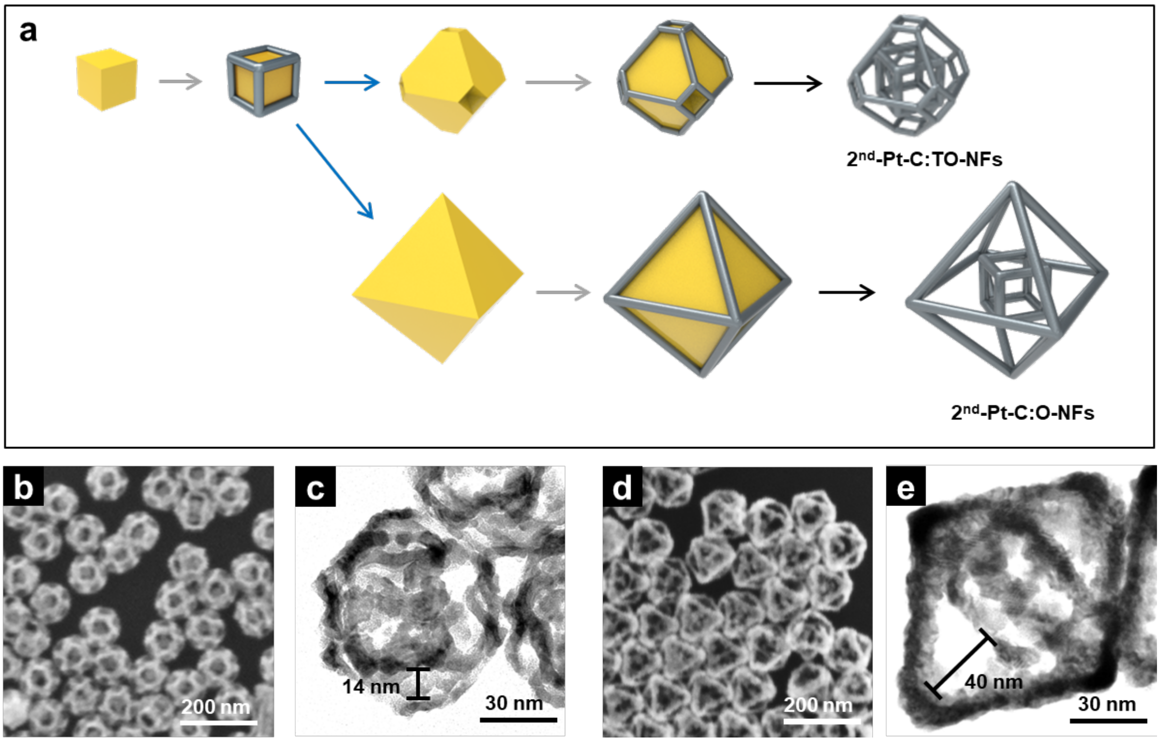

Supplementary Fig. 11 | Controlling the outer shape of nanoframes of 2nd nanoframe. (a) Schematic illustration of synthetic pathways for 2nd nanoframes with different outer shapes. SEM image of (b and c) 2nd-Pt-C:TO-NFs and (d and e) 2nd-Pt-C:O-NFs.

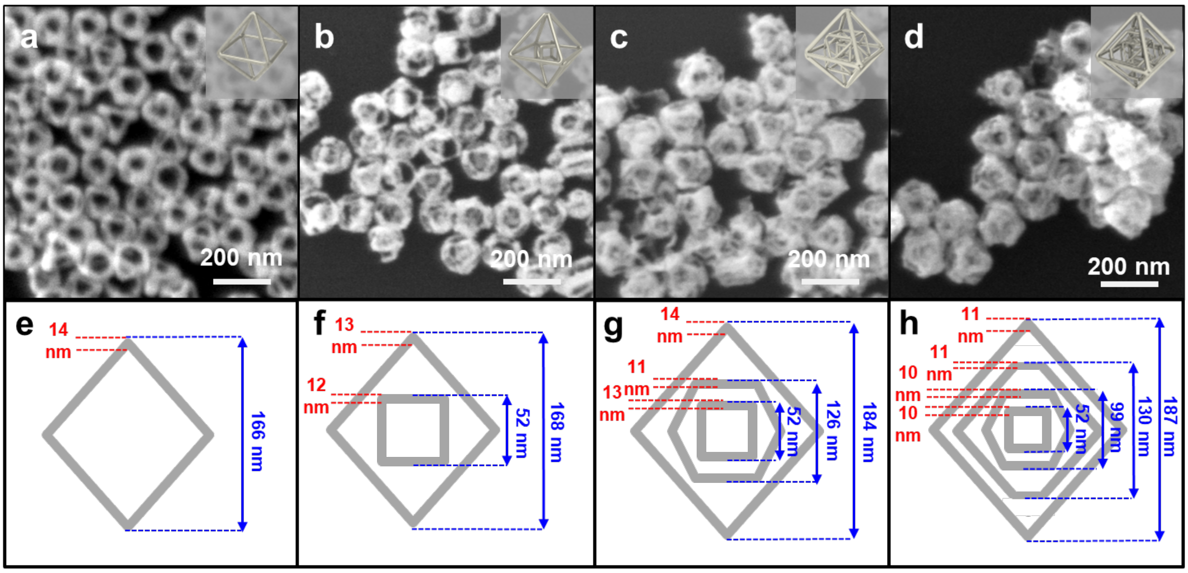

Supplementary Fig. 12 | Synthesis of complex Ag nanoframes. (a to d) SEM images of complex Ag nanoframe with different numbers of frames and (e to h) their corresponding dimension information.

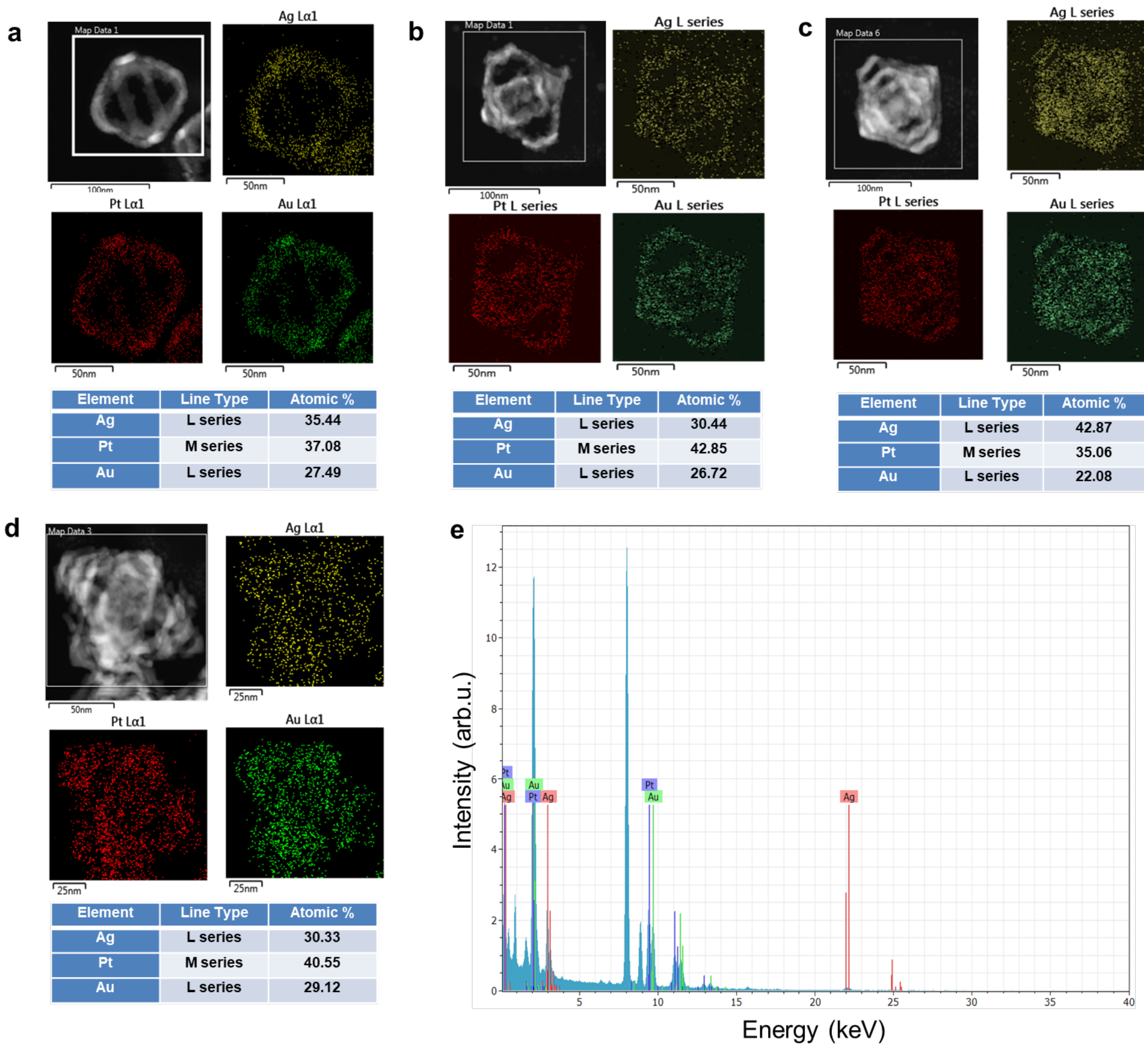

Supplementary Fig. 13 | Analysis of element distribution of complex Ag nanoframes with different numbers of nanoframes. (a to d) HAADF-STEM images and EDS image mapping data of (a) 1st Ag nanoframes, (b) 2nd Ag nanoframes, (c) 3rd Ag nanoframes, and (d) 4th Ag nanoframes. (e) EDS spectrum corresponding to 4th Ag nanoframes. Each color used in EDS mapping images: yellow for Ag, red for Pt, and green for Au, respectively.

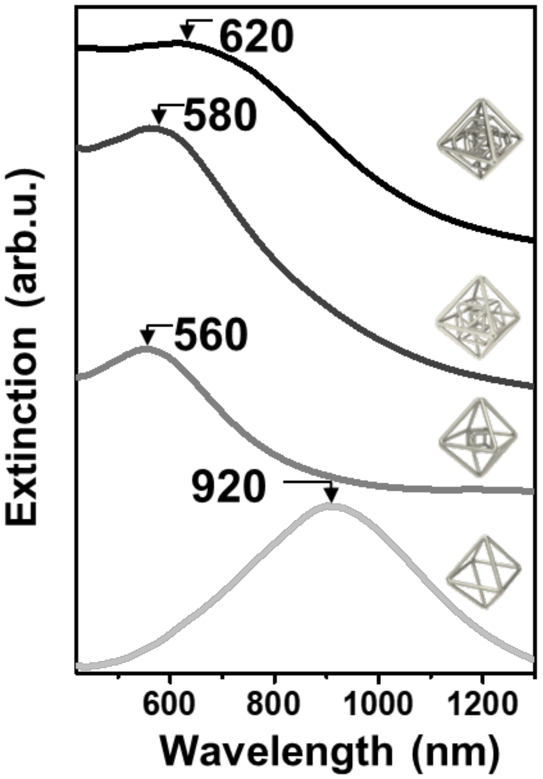

Supplementary Fig. 14 | Theoretically calculated UV-vis-NIR spectrum for Ag nanoframes with different number of nanoframes through FEM method.

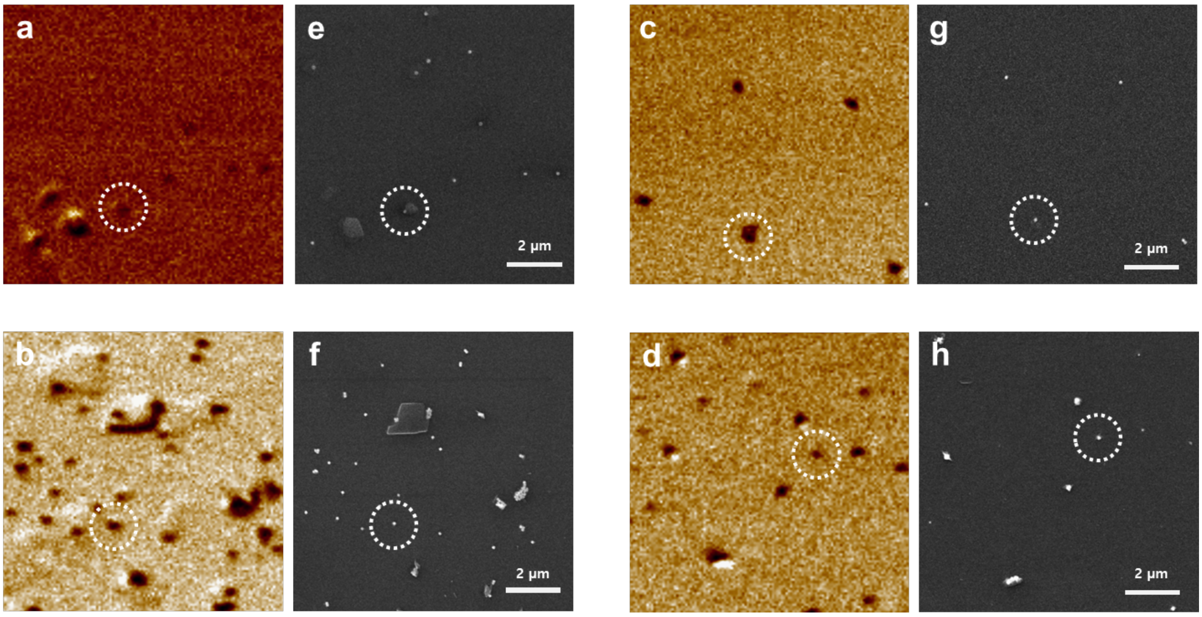

Supplementary Fig. 15 | Confirming the position of single particle of complex Ag nanoframes. (a to h) Rayleigh scattering images and the corresponding SEM images of (a and e) 1st, (b and f) 2nd, (c and g) 3rd, and (d and h) 4th Ag nanoframes. The dotted circles represent the single nanoparticles described in Fig. 5.

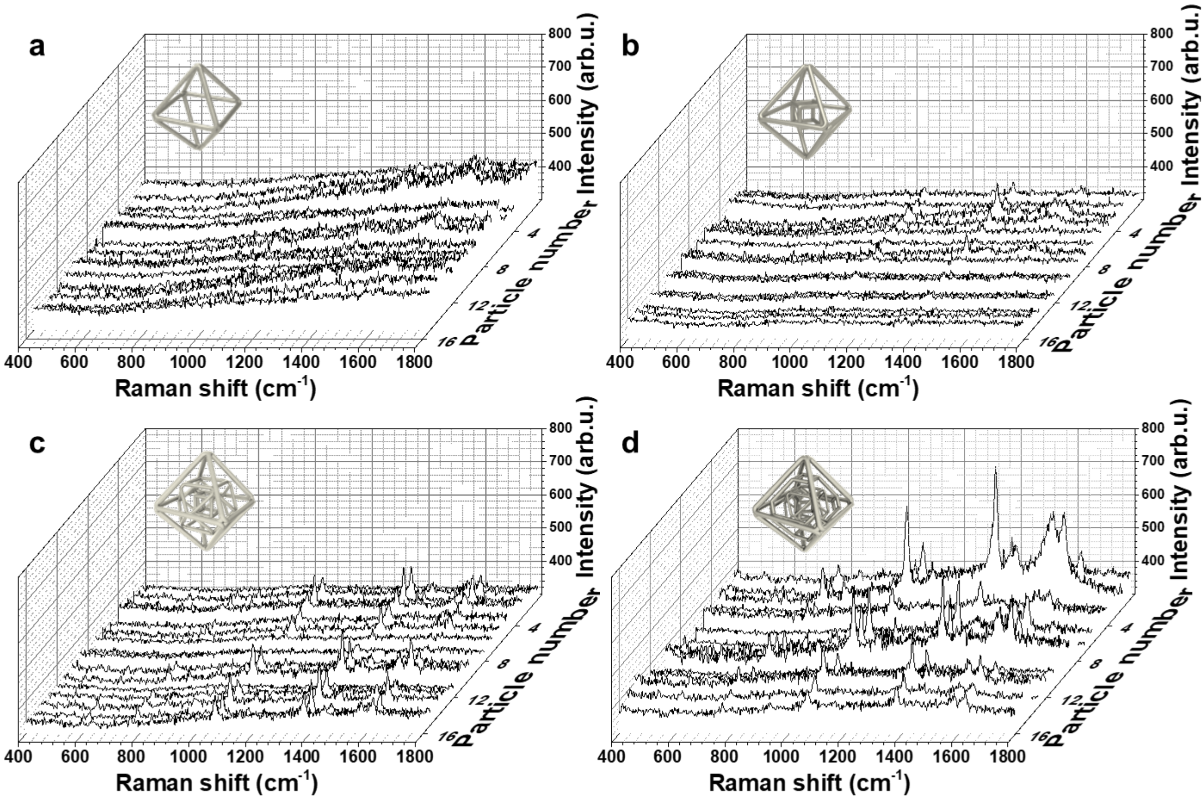

Supplementary Fig. 16 | Single-particle SERS data of complex Ag nanoframes with different numbers of frames (532 nm laser excitation). (a) Raman signal of 1st Ag nanoframes, (b) Raman signal of 2nd Ag nanoframes, (c) Raman signal of 3rd Ag nanoframes, and (d) Raman signal of 4th Ag nanoframes.

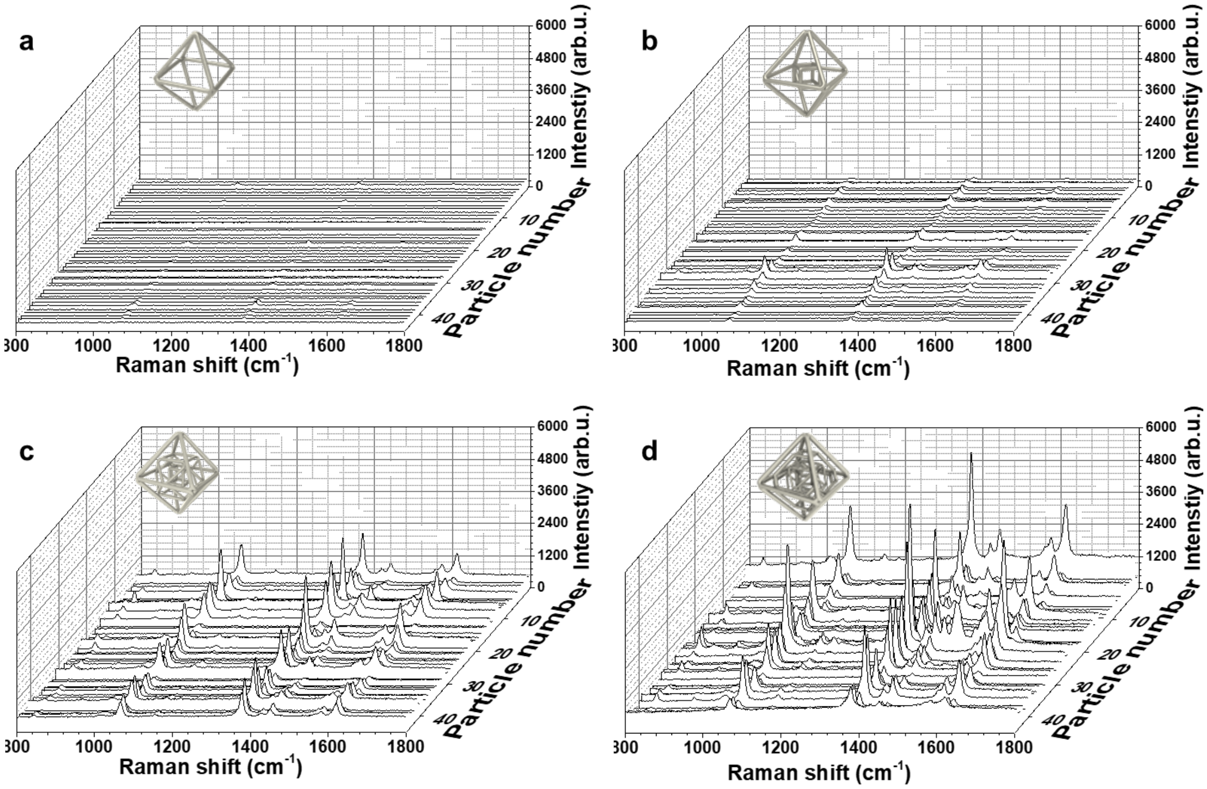

Supplementary Fig. 17 | Single-particle SERS data of complex Ag nanoframes with different numbers of frames (633 nm laser excitation). (a) Raman signal of 1st Ag nanoframes, (b) Raman signal of 2nd Ag nanoframes, (c) Raman signal of 3rd Ag nanoframes, and (d) Raman signal of 4th Ag nanoframes.

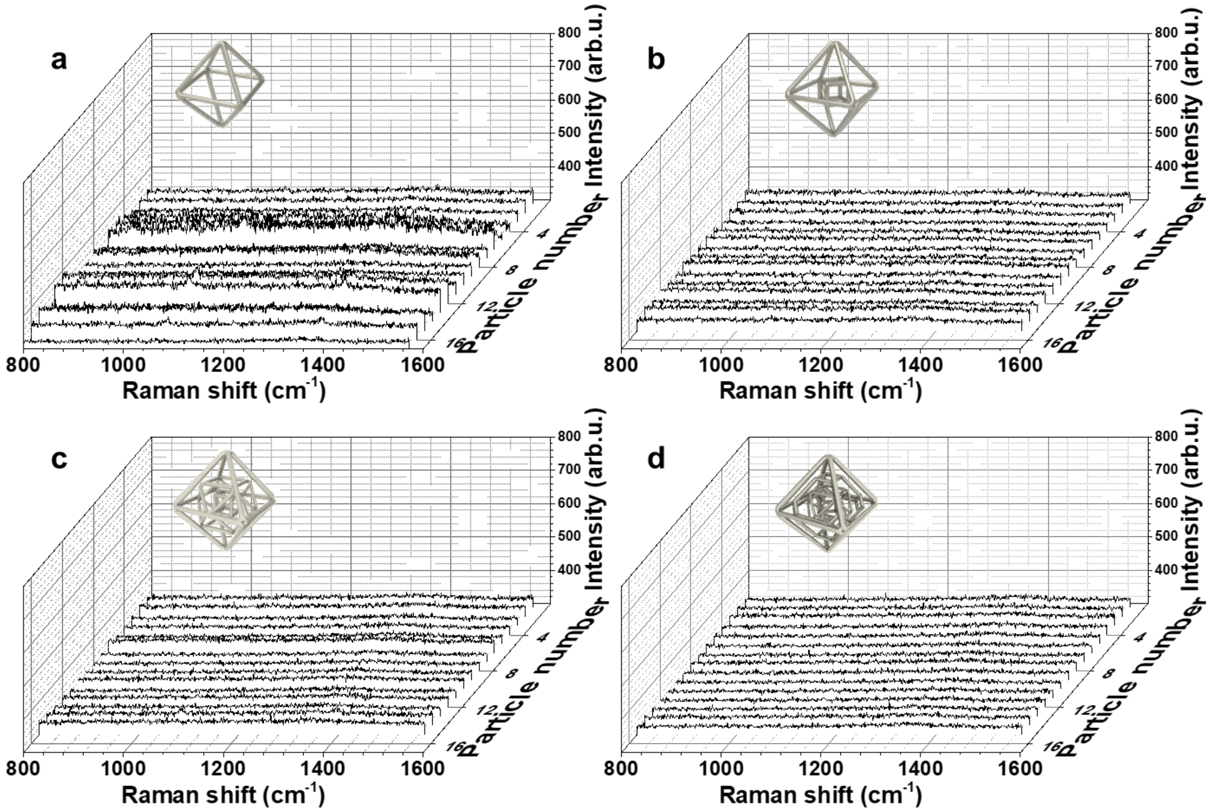

Supplementary Fig. 18 | Single-particle SERS data of complex Ag nanoframes with different numbers of frames (785 nm laser excitation). (a) Raman signal of 1st Ag nanoframes, (b) Raman signal of 2nd Ag nanoframes, (c) Raman signal of 3rd Ag nanoframes, and (d) Raman signal of 4th Ag nanoframes.

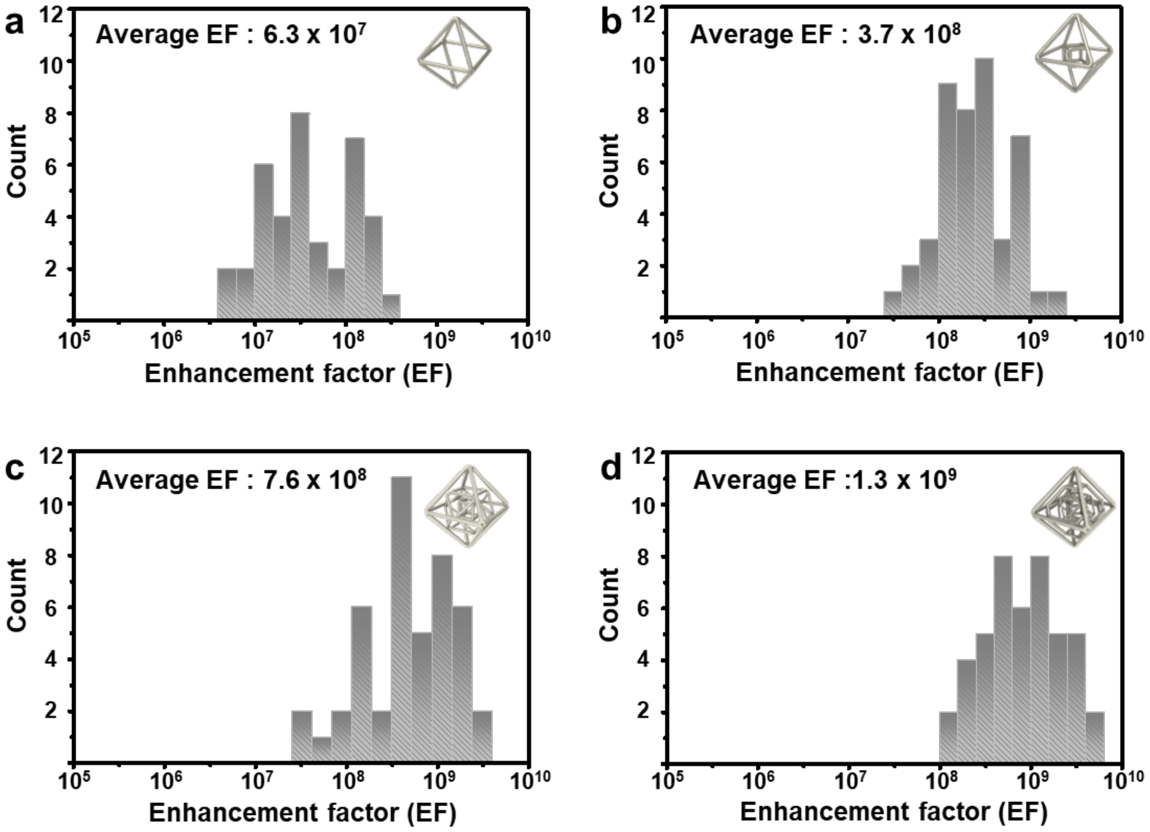

Supplementary Fig. 19 | Calculated values of the enhancement factor. Enhancement factor (EF) of (a) 1st, (b) 2nd, (c) 3rd, and (d) 4th Ag nanoframes.

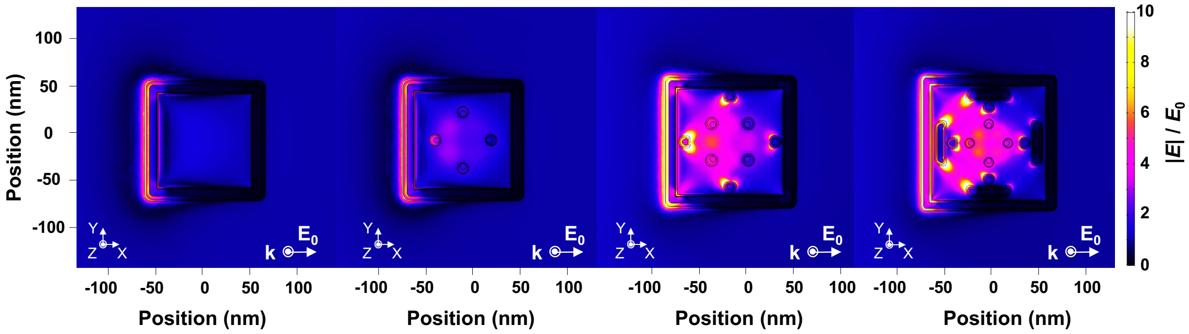

Supplementary Fig. 20 | Electromagnetic near-field contour maps (in the XY cross-section) of complex Ag nanoframes for different numbers of nanoframes (single to quadruple) obtained by a FEM simulation (Excitation wavelength: 633 nm).

Supplementary Table. 1 | ICP-MS analysis of 1st, 2nd, 3rd, and 4th Ag nanoframes.

	1st-Ag-O-NF	2nd-Ag-O:O-NF	3rd-Ag-TO:TO:O-NF	4th-Ag-C:CO:TO:O-NF
**Pt content (%)**	51	48	44	51
**Au content (%)**	27	32	37	29
**Ag content (%)**	22	20	19	20

Supplementary Table 2 | EF comparison of previously reported Ag nanomaterials.

Type of nanoparticles	Raman analytes	Excitation wavelength	Enhancement factor (EF)	References
**Au Ag alloy nanourchins**	Crystal violet	633 nm	$\sim 10^{9}$	1
**Au-Ag core-shell nanodumbells**	Cy3 dye	514 nm	$\sim 10^{13}$	2
**Anisotropic Ag nanoparticles**	Benzenethiol	514, 633, 785 nm	3×10^{4}, 5×10^{4}	3
**Ag double nanorings**	2-Naphthalenthiol	785 nm	1.4×10^{8}, 2.6×10^{8}, 5.1×10^{8}	4
**Ag nanoplates with ultranarrow gaps**	2-Naphthalenthiol	785 nm	4.1×10^{10}	5
**Porous AuAg nanoparticles**	Crystal violet	633 nm	10^{7}	6
**Dimer of Ag nanoparticles**	Rhodamine 6G	500 nm	1.4×10^{9}	7
**Ag nanoparticles**	4-aminobenzenethiol	632 nm	5.0×10^{5}	8
**Ag shell – Au satellite (Ag – Au SS)**	4-fluorobenzenethiol	785 nm	1.4×10^{6}	9
**bimetallic (Au/Ag) hierarchical structure**	2-naphthalenethiol	633 nm	2×10^{7}	10
**Au@Ag core–shell nanocubes**	4-Mercaptobenzoic acid	785 nm	2.2×10^{6}	11
**silver core-gold shell nanostructures**	alkanethiol, 11-mercaptoundecanoic acid	633 nm	6.51×10^{5}	12
**Ag@Au nanowire**	4-nitrothiophenol	532 nm	1.3×10^{9}	13
**N-th nanoframes**	2-Naphthalenthiol	633 nm	1.3×10^{9}	This work

Supplementary Table 3 | Added reagents volume for Pt deposition on Au nanoparticles in synthetic process of 3rd-Pt-TO:TO:O-NF.

Order	50 mM CTAB (mL)	2 mM $AgNO_3$ (µL)	0.1 M AA and HCl (µL)	1 mM H_2PtCl_6 (µL)	Optical density, wavelength of resulting Au@Pt NPs
**1st (A)**	2.5	6.25	48 and 48	62.5	0.8, 605 nm
**2nd (C)**	4	10.4	96 and 96	104	0.9, 661 nm
**3rd (E)**	4	9.6	96 and 96	96	0.6, 676 nm

Supplementary Table 4 | Added reagents volume for well-faceted overgrowth of Au on Au@Pt nanoparticles in synthetic process of 3rd-Pt-TO:TO:O-NF.

Order	0.2 M CTAC volume (mL)	2 mM $AgNO_3$ volume (µL)	0.1 M AA volume (µL)	2 mM $HAuCl_4$ volume (µL)
**2nd (B)**	3	90	9	90
**3rd (D)**	3	150	15	150

Supplementary Table 5 | Added reagents volume for Pt deposition on Au nanoparticles in synthetic process of 4th-Pt-C:CO:TO:O-NF.

Order	0.1 M CTAB (mL)	2 mM $AgNO_3$ (µL)	0.1 M AA and HCl (µL)	1 mM H_2PtCl_6 (µL)	Optical density, wavelength of resulting Au@Pt NPs
**1st (F)**	2.5	6.25	50 and 95	62.5	1.0, 637 nm
**2nd (H)**	4	2.3	48 and 48	46	0.9, 610 nm
**3rd (J)**	4	3.8	48 and 48	76	0.8, 677 nm
**4th (L)**	4	4	48 and 48	80	0.8, 769 nm

Supplementary Table 6 | Added reagents volume for well-faceted overgrowth of Au on Au@Pt nanoparticles in synthetic process of 4th-Pt-C:CO:TO:O-NF.

Order	0.2 M CTAC volume (mL)	2 mM $AgNO_3$ volume (µL)	0.1 M AA volume (µL)	2 mM $HAuCl_4$ volume (µL)
**2nd (G)**	3	120	12	120
**3rd (I)**	3	150	15	150
**4th (K)**	3	220	22	220

Supplementary References

1. Liu, Z. et al. Highly sensitive, uniform, and reproducible surface-enhanced Raman spectroscopy from hollow Au-Ag alloy nanourchins. Adv. Mater. 26, 2431–2439 (2014).
2. Lee, J. H. et al. Tuning and maximizing the single-molecule surface-enhanced Raman scattering from DNA-tethered nanodumbbells. ACS Nano 6, 9574–9584 (2012).
3. Mulvihill, M. J., Ling, X. Y., Henzie, J. & Yang, P. Anisotropic etching of silver nanoparticles for plasmonic structures capable of single-particle SERS. J. Am. Chem. Soc. 132, 268–274 (2010).
4. Yoo, S. et al. Silver Double Nanorings with Circular Hot Zone. J. Am. Chem. Soc. 142, 12341-12348 (2020).
5. Jiang, T. et al. Construction of Long Narrow Gaps in Ag Nanoplates. J. Am. Chem. Soc. 140, 15560–15563 (2018).
6. Liu, K. et al. Porous Au-Ag nanospheres with high-density and highly accessible hotspots for SERS analysis. Nano Lett. 16, 3675–3681 (2016).
7. Camden, J. P. et al. Probing the structure of single-molecule surface-enhanced Raman scattering hot spots. J. Am. Chem. Soc. 130, 12616–12617 (2008).
8. Kim, K., Choi, J. Y., Lee, H. B. & Shin, K. S. Raman scattering of 4-aminobenzenethiol sandwiched between Ag nanoparticle and macroscopically smooth Au substrate: Effects of size of Ag nanoparticles and the excitation wavelength. J. Chem. Phys. 135, 124705 (2011).
9. Chang, H. et al. Ag shell-Au satellite hetero-nanostructure for ultra-sensitive, reproducible, and homogeneous NIR SERS activity. ACS Appl. Mater. Interfaces 6, 11859–11863 (2014).
10. Fu, C. Y., Kho, K. W., Dinish, U. S., Koh, Z. Y. & Malini, O. Enhancement in SERS intensity with hierarchical nanostructures by bimetallic deposition approach. J. Raman Spectrosc. 43, 977–985 (2012).
11. Liu, Y. et al. Au@Ag core-shell nanocubes: Epitaxial growth synthesis and surface-enhanced Raman scattering performance. Phys. Chem. Chem. Phys. 17, 6819–6826 (2015).
12. Raveendran, J. & Docoslis, A. Detection and quantification of toxicants in food and water using Ag–Au core-shell fractal SERS nanostructures and multivariate analysis. Talanta 231, 122383 (2021).
13. Zhu, Y., Tang, H., Wang, H. & Li, Y. In Situ SERS Monitoring of the Plasmon-Driven Catalytic Reaction by Using Single Ag@Au Nanowires as Substrates. Anal. Chem. 93, 11736–11744 (2021).
