# Supplementary material for: Nesting of multiple polyhedral plasmonic nanoframes into a single entity
Source: Nat Commun. 2022 Aug 4;13:4544. doi: 10.1038/s41467-022-32261-9 (PMC9352762; doi:10.1038/s41467-022-32261-9)
Supplement: Supplementary file 3 — Description of Additional Supplementary Files [file 41467_2022_32261_MOESM3_ESM.pdf]

<u>Description of Additional Supplementary Files</u>

Supplementary Movie 1 | Tilt series of TEM images (taken from -60 ° to + 60 °) of 2nd-PtO:O-NF.

Supplementary Movie 2 | Tilt series of TEM images (taken from -60 ° to + 60 °) of 3rd-PtTO:TO:O-NF.

Supplementary Movie 3 | Tilt series of TEM images (taken from -60 ° to + 60 °) of 4th-PtC:CO:TO:O-NF.

Supplementary Movie 4 | Tilt series of TEM images (taken from -60 ° to + 60 °) of 4th-AgC:CO:TO:O-NF.
